# Visual and Predictive Assessment of Pneumothorax Recurrence in Adolescents Using Machine Learning on Chest CT

**DOI:** 10.3390/jcm14175956

**Published:** 2025-08-23

**Authors:** Kwanyong Hyun, Jae Jun Kim, Kyong Shil Im, Sang Chul Han, Jeong Hwan Ryu

**Affiliations:** 1 Department of Thoracic and Cardiovascular Surgery, St. Vincent’s Hospital, College of Medicine, The Catholic University of Korea, Seoul 06591, Republic of Korea; pipedragon@gmail.com; 2 Department of Thoracic and Cardiovascular Surgery, Uijeongbu St. Mary’s Hospital, College of Medicine, The Catholic University of Korea, Seoul 06591, Republic of Korea; 3 Department of Anesthesiology and Pain Medicine, Uijeongbu St Mary’s Hospital, College of Medicine, The Catholic University of Korea, Seoul 06591, Republic of Korea; donga@catholic.ac.kr (K.S.I.); josephhan12@naver.com (S.C.H.); wjdghks7706@naver.com (J.H.R.)

**Keywords:** spontaneous pneumothorax, prediction, machine learning, chest computed tomography

## Abstract

**Background:** Spontaneous pneumothorax (SP) in adolescents has a high recurrence risk, particularly without surgical treatment. This study aimed to predict recurrence using machine learning (ML) algorithms applied to chest computed tomography (CT) and to visualize CT features associated with recurrence. **Methods:** We retrospectively reviewed 299 adolescents with conservatively managed SP from January 2018 to December 2022. Clinical risk factors were statistically analyzed. Chest CT images were evaluated using ML models, with performance assessed by AUC, accuracy, precision, recall, and F1 score. Gradient-weighted Class Activation Mapping (Grad-CAM) was used for visual interpretation. **Results:** Among 164 right-sided and 135 left-sided SP cases, recurrence occurred in 54 and 43 cases, respectively. Mean recurrence intervals were 10.5 ± 9.9 months (right) and 12.7 ± 9.1 months (left). Presence of blebs or bullae was significantly associated with recurrence (*p* < 0.001). Neural networks achieved the best performance (AUC: 0.970 right, 0.958 left). Grad-CAM confirmed the role of blebs/bullae and highlighted apical lung regions in recurrence, even in their absence. **Conclusions:** ML algorithms applied to chest CT demonstrate high accuracy in predicting SP recurrence in adolescents. Visual analyses support the clinical relevance of blebs/bullae and suggest a key role of apical lung regions in recurrence, even when blebs/bullae are absent.

## 1. Introduction

Pneumothorax is defined as the presence of air within the pleural cavity, disrupting the normal negative pressure and potentially leading to partial or total lung collapse [1]. Common symptoms include chest pain and dyspnea [2]. Spontaneous pneumothorax (SP), a subset of pneumothorax, is further categorized into primary and secondary types [1]. Primary SP typically occurs in young, otherwise healthy individuals without underlying lung disease, whereas secondary SP develops in the presence of pre-existing pulmonary conditions, requiring distinct treatment approaches [2,3]. Surgical indications for SP generally include the identification of definitive blebs or bullae on imaging studies, massive or persistent air leaks lasting several days, recurrent pneumothorax, concurrent hemothorax, and high-risk occupational factors [1,3,4]. However, these indications are applied variably depending on individual patient circumstances [4,5]. For instance, elderly patients with secondary SP who meet surgical criteria are often deemed unsuitable for surgery due to severe underlying pulmonary dysfunction or comorbidities [1,4].

The failure to identify or resect blebs or bullae during surgery is the most significant independent predictor of SP recurrence, with postoperative recurrence rates reported at approximately 5% [1,6]. Thus, the presence of blebs or bullae remains a critical concern for surgeons when selecting surgical strategies and implementing measures to minimize recurrence risk [3,7]. In contrast, adolescents with primary SP who are managed conservatively experience significantly higher recurrence rates—ranging from 20% to 50% within the first year after the initial episode—even when definitive blebs or bullae are not detected on chest computed tomography (CT) [1,3,4,5,8]. This high recurrence rate and its variability underscore the need for robust prediction methods and strategies to effectively manage SP recurrence in clinical practice [1,3]. Although the association between blebs or bullae and SP recurrence is well-recognized in clinical practice, imaging-based studies directly verifying this relationship remain limited [6,9]. To address these gaps, this study aims to achieve the following: (1) utilize machine learning algorithms to predict primary SP recurrence in non-surgical adolescent cases; (2) visually verify the association between SP recurrence and blebs or bullae identified on chest CT; and (3) explore the role of apical lung regions in SP recurrence among cases without definitive blebs or bullae through visual verification. This article is presented in accordance with the TRIPOD reporting checklist.

## 2. Materials and Methods

**Figure 1:**
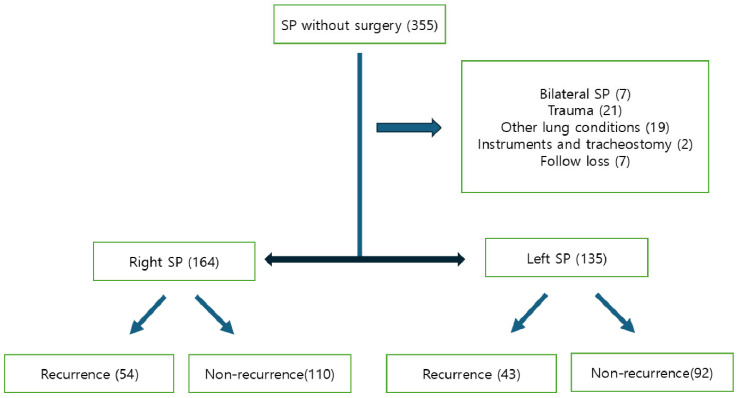
Flowchart showing the selection and classification of adolescent patients with spontaneous pneumothorax (SP) managed conservatively without surgery. Patients were excluded for bilateral SP, trauma-related etiology, other pulmonary conditions, tracheostomy or instrumentation, or loss to follow-up. The remaining cohort was stratified by laterality (right or left SP) and further categorized by recurrence status.

All consecutive adolescent patients (aged 13 to 20 years) who were managed conservatively for primary SP at Uijeongbu Saint Mary’s Hospital between January 2018 and December 2022 were retrospectively reviewed (Figure 1). Chest CT scans, performed without contrast enhancement, were routinely conducted during the initial episode to aid in evaluation and selection of appropriate management strategies. Conservative treatments included oxygen therapy and closed thoracostomy drainage without pleurodesis [3,4]. In this study, surgical intervention for SP was considered when patients demonstrated definitive blebs or bullae on chest CT; experienced massive or persistent air leakage lasting several days; had recurrent episodes of SP; presented with clinically significant hemothorax or tension pneumothorax; or developed simultaneous bilateral SP [1,9]. All procedures were carried out using thoracoscopic approaches. Cases were excluded if they had any underlying lung conditions, including concurrent bilateral SP, primary or metastatic pulmonary cancer, catamenial pneumothorax, tuberculosis-induced lung lesions, pneumonia (such as COVID-19 infection), or traumatic pneumothorax. Following the completion of the initial treatment for spontaneous pneumothorax (SP), recurrence was defined as its reappearance. To mitigate the influence of time-related factors, the follow-up period was restricted to two years following treatment as this is the timeframe during which recurrence is most likely [1,3].

Parameters considered to be associated with SP recurrence in the peri-management data analysis included age at the first episode, sex, smoking history, and the identification of definitive blebs or bullae in the apical regions on chest CT [1,2]. Blebs or bullae were regarded as definitive when identified as clearly defined, well-demarcated air-filled lesions greater than 1 cm on chest CT scans [7]. Some patients refused surgery due to personal circumstances, such as fear of the surgery, lack of time, or early cessation of air leaks, even when definitive blebs or bullae were present. Consequently, some patients with definitive blebs or bullae were included in this study. Since chest CT image data were used, all data were analyzed separately for the right and left sides.

### 2.1. Statistical Analysis

All data in this study are presented as the mean ± standard deviation (SD). Group comparisons were performed with Student’s *t*-tests, and chi-square tests were used for categorical variables to assess their independence. Binary logistic regression with a backward stepwise method was used for both univariate and multivariate analyses to identify independent influencing factors. The statistical analyses were conducted using SPSS software (version 22.0; IBM Corp., Armonk, NY, USA). A *p*-value of less than 0.05 was considered to be statistically significant.

### 2.2. Machine Learning Algorithms

Image analysis can be challenging but machine learning is highly effective, particularly excelling in classification and prediction tasks [10]. By leveraging advanced algorithms, machine learning can identify patterns, extract important features, and precisely classify images [11,12]. Such capabilities allow machine learning models to generate reliable predictions from image data, thereby enhancing both efficiency and precision, especially in medical applications [11,12]. Therefore, machine learning analyses were employed in this study to predict SP recurrence using chest CT scan images. The CT scan images that best displayed blebs or bullae in the apical lung were selected for machine learning analysis to ensure the utilization of the most clearly visible regions. Additionally, in cases where no definitive blebs or bullae were identified, CT scan images showing the most prominent dystrophic changes in the apical regions were selected for analysis [13].

Machine learning analyses in this study were performed with the Orange^®^ data mining program (version 3.37), an open-source toolkit created by the Bioinformatics Lab at the University of Ljubljana, Slovenia. This program offers a user-friendly visual interface and supports Python (version 3.13.0; Python Software Foundation, Wilmington, DE, USA) scripting for more advanced users [14]. For image classification and feature extraction within Orange^®^, Google’s Inception v3, a deep learning model, was used as the image embedder [15]. The model was trained and validated using a 10-fold stratified cross-validation approach. Model performance was assessed using several metrics, including area under the curve (AUC), accuracy, precision, F1 score, and recall, with corresponding 95% confidence intervals calculated as previously recommended in machine learning studies for medical imaging and outcome prediction [16,17]. The best-performing model was chosen based on its AUC, which indicates its ability to differentiate between classes. The five algorithms used for prediction were gradient boosting (GB), logistic regression (LR), K-nearest neighbors (KNN), random forest (RF), and neural networks (NN). The GB model used 100 trees, a learning rate of 0.100, a maximum tree depth of three, and two samples per split. Ridge (L2) regularization with C = 1 was applied to the LR model. The KNN model was set with five neighbors, using Euclidean distance and uniform weights. The RF model was composed of ten trees with a minimum split size of five. The NN model had hidden layers of 100, 50, and 20 neurons, and utilized rectified linear unit (ReLU) activation with Adam optimization (α = 0.0001, 200 iterations).

Compared to conventional image analysis, which typically requires a large dataset, this study had a limited number of images. To address this, we employed data augmentation—specifically rotation—and transfer learning techniques to enhance the model’s performance [12,15,18]. Data augmentation enhances the size and diversity of training datasets by generating modified versions of existing data or synthesizing new samples using deep learning techniques [12]. This process introduces variations to simulate real-world scenarios, reducing overfitting and improving model accuracy, particularly when datasets are small or imbalanced [12]. It offers a cost-effective alternative to extensive data collection and manual annotation, strengthening model generalization to unseen data [12]. Transfer learning leverages knowledge from one domain to improve model performance in a related domain [12]. By reusing features or weights from pre-trained models, it addresses challenges like limited data and high computational demands [12,15]. Google’s Inception v3, a widely used pre-trained convolutional neural network, is based on the ImageNet dataset, containing millions of labeled images [15]. Its efficient architecture and advanced features make it a popular choice for applications such as medical image classification [15]. Fine-tuning involves retaining lower-level layers for general patterns while adapting higher-level layers to the target task, enabling rapid training and better generalization [12,15,19].

Additionally, Gradient-weighted Class Activation Mapping (Grad-CAM) in conjunction with transfer learning based on the Google’s Inception v3 model was employed to visualize the association between SP recurrence and the presence of blebs or bullae on chest CT scan images [20]. Using Grad-CAM, heatmaps were generated to highlight the regions of the images most influential to the model’s predictions [20]. These visualizations revealed that the model primarily focused on clinically relevant lung regions, enhancing both the interpretability and the potential clinical utility of our predictive model [20]. The Grad-CAM algorithm was implemented using Google Colab^®^ (Google Research, Mountain View, CA, USA), a cloud-based platform that enables the execution of Python code within a browser-based notebook. This platform is frequently employed for data science and machine learning tasks as it offers complementary access to high-performance computing resources, including graphics processing units (GPUs) and tensor processing units (TPUs) [21].

### 2.3. Ethical Statement

Informed consent was waived for this study because of its retrospective design, which did not necessitate the disclosure of patient information. This research was reviewed and approved by the Uijeongbu Saint Mary’s Hospital Ethics Committee (Approval No. UC25RISI0012) on 7 April 2025. Furthermore, this study was conducted in accordance with the ethical principles of the revised 2013 Declaration of Helsinki.

## 3. Results

This study included 299 consecutive cases of pneumothorax, comprising 164 right-sided and 135 left-sided cases. Patients presenting with a first episode of right-sided SP had a mean age of 17.2 ± 1.5 years, while those with left-sided SP had a mean age of 17.5 ± 1.5 years. This study included 280 male and 19 female patients. The mean observation times were 13.7 ± 10.5 months for the right side and 15.7 ± 9.6 months for the left side. A total of 54 right-sided and 43 left-sided cases of spontaneous pneumothorax (SP) experienced a recurrence. The mean interval to recurrence was 10.5 ± 9.9 months for right-sided SP and 12.7 ± 9.1 months for left-sided SP. The general clinical profiles of the study cohort are provided in Table 1.

### 3.1. Risk Factors for Recurrence in Young Adolescent Patients

Numerous studies have identified factors associated with SP recurrence, including age, sex, chronic obstructive pulmonary disease (COPD), smoking history, and management options (non-surgical vs. surgical) [1,22,23]. Among these, surgery, specifically the resection of blebs or bullae, is widely recognized as the most significant independent factor influencing SP recurrence [1,6]. In this study, none of the cases involved surgical intervention. Only the presence of definitive blebs or bullae was significantly associated with SP recurrence in the multivariate analysis (Table 2).

### 3.2. Prediction of SP Recurrence Using Machine Learning Models with Chest CT Imaging

Following data augmentation techniques, we obtained a total of 984 right-sided images (324 recurrence and 660 non-recurrence) and 810 left-sided images (258 recurrence and 552 non-recurrence). Since the presence of definitive blebs or bullae was significantly associated with SP recurrence in both univariate and multivariate analyses in our cohort, we utilized chest CT imaging data in Orange^®^ and applied five machine learning algorithms to predict SP recurrence.

**Figure 2:**
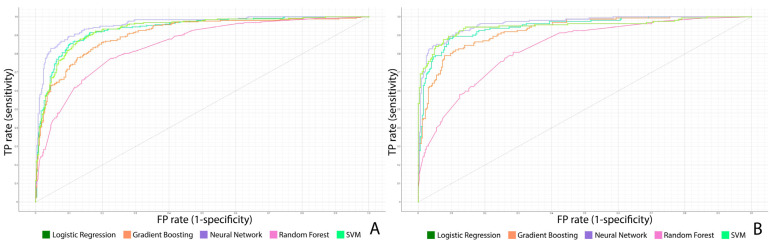
Receiver operating characteristic (ROC) curves for prediction of spontaneous pneumothorax recurrence: (**A**) shows results for right-sided pneumothorax, and (**B**) for left-sided cases. TP rate = true positive rate; FP rate = false positive rate.

In the prediction of SP recurrence, the neural network (NN) model emerged as the top-performing algorithm for both lung laterality groups. On the right side, the NN model yielded an AUC of 0.970 (95% CI: 0.961–0.979), accompanied by an accuracy of 0.937, an F1 score of 0.936, a precision of 0.937, and a recall of 0.937. Similarly, for the left side, the NN model was the most effective, achieving an AUC of 0.958 (95% CI: 0.949–0.967), an accuracy of 0.905, and matching F1 score, precision, and recall values of 0.905 (Table 3). Receiver operating characteristic (ROC) curves for predicting SP recurrence are shown in Figure 2.

To evaluate the possibility of predicting SP recurrence in cases without definitive blebs or bullae, patients were divided into two groups (those with definitive blebs or bullae and those without) and the analysis was conducted accordingly. The performance of different machine learning models varied depending on the presence of definitive blebs or bullae. For cases with these lesions, the SVM model demonstrated superior performance on the right side (AUC: 0.997; accuracy: 0.979; F1 score: 0.979; precision: 0.979; and recall: 0.979), while the LR model was most effective on the left side (AUC: 0.989; accuracy: 0.931; F1 score: 0.927; precision: 0.937; and recall: 0.931). In the absence of definitive blebs or bullae, LR was the best-performing model for the right side (AUC: 0.980; accuracy: 0.939; F1 score: 0.938; precision: 0.939; and recall: 0.939). For left-sided cases without these lesions, the NN model achieved the highest performance metrics, with an AUC of 0.959 and an accuracy of 0.928.

### 3.3. Grad-CAM

**Figure 3:**
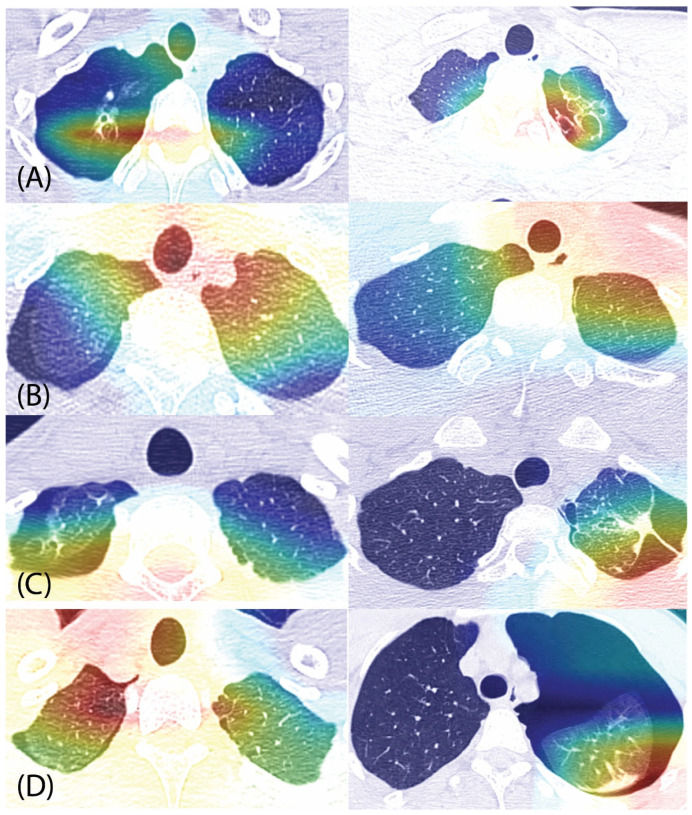
Gradient-weighted Class Activation Mapping (Grad-CAM) visualizations highlighting chest CT regions associated with recurrence of primary spontaneous pneumothorax (SP). Apical lung regions were frequently activated in cases with recurrence, particularly in the presence of definitive blebs or bullae. Notably, in recurrent cases without identifiable blebs or bullae, apical activation was still observed, suggesting a potential subclinical contribution. Panels: (**A**) recurrence with blebs or bullae; (**B**) recurrence without blebs or bullae; (**C**) non-recurrence with blebs or bullae; (**D**) non-recurrence without blebs or bullae.

The primary cause of SP development is the rupture of blebs or bullae in the lung apex, and the presence of these lesions is a main factor contributing to SP recurrence. However, few imaging studies using chest CT have been conducted to directly visualize this phenomenon [11,18]. The Grad-CAM algorithm was used in this study to visualize the relationship between SP recurrence and definitive blebs or bullae, specifically by highlighting the image regions most strongly associated with recurrence. Additionally, in recurrent cases without definitive blebs or bullae, we aimed to highlight the image regions most closely associated with SP recurrence [7]. Grad-CAM analysis revealed that definitive blebs or bullae in the lung apex were strongly associated with SP recurrence. Furthermore, in both recurrent and non-recurrent cases without definitive blebs or bullae, the apical regions were identified as playing a critical role in SP recurrence (Figure 3).

## 4. Discussion

For surgeons, the identification and resection of blebs or bullae are essential in the management of SP as their presence is the leading cause of recurrence [7,24]. In primary SP, surgery is typically recommended when definitively visible blebs or bullae exist, and postoperative recurrence rates are reported to be approximately 5% [1,6]. In contrast, patients—particularly adolescents without definitive blebs or bullae or those with tiny ones—who are treated conservatively experience significantly higher recurrence rates, ranging from 20% to 50% within the first year after the initial episode [1,3,25]. These findings underscore the importance of identifying and addressing blebs or bullae that contribute to recurrence in order to select the appropriate surgical approach.

Accurately predicting recurrence in SP cases is crucial for guiding clinical decision-making and improving patient outcomes [26]. Previous studies have shown that SP recurrence is influenced by the presence of blebs or bullae, which can evolve over time, particularly in growing adolescent patients [1,6]. Understanding the interplay of these is essential for accurately predicting the likelihood of SP recurrence. Consistent with previous studies, the present study demonstrated that SP recurrence in young adolescent patients is associated with the presence of definitive blebs or bullae [3,6]. Therefore, analyzing both the presence and characteristics of blebs or bullae is essential [7]. Historically, chest CT scans have been the primary tool for studying SP recurrence, providing insights into the characteristics of blebs or bullae and apical dystrophic changes in the lung, which are considered to be associated with SP recurrence [1,27,28,29]. The presence of definitive blebs or bullae, as well as the potential for SP recurrence due to their future formation or the presence of tiny ones, are key concerns in the management of young adolescent SP patients [6,7,30]. Deep learning-based machine learning algorithms have demonstrated strong performance in analyzing these factors [11,12]. The present study demonstrated that machine learning algorithms achieved excellent performance in predicting SP recurrence in non-surgical cases, both with and without definitive blebs or bullae. This finding suggests that more invasive management strategies may be recommended even in cases without definitive blebs or bullae, including those with tiny lesions, due to the high risk of recurrence.

While the association between blebs or bullae and SP recurrence is well-established and widely recognized in clinical practice, there is a lack of imaging-based studies that directly verify this association or predict SP recurrence due to their future formation or the presence of tiny lesions [7,27]. In this study, the association between blebs or bullae and SP recurrence was visually shown; furthermore, in cases without definitive blebs or bullae, the apical regions were identified as playing a critical role in SP recurrence, even in the absence of clearly visible abnormalities, suggesting the potential to predict the future formation of blebs or bullae contributing to recurrence.

A robust predictive model for SP recurrence serves a dual function: it should both identify the underlying risk factors and quantify the likelihood of recurrence [14]. This dual functionality provides clinicians with a deeper understanding of individual patient risks, which in turn facilitates the development of more personalized and preventative management strategies aimed at improving overall patient outcomes. In addition, including 95% confidence intervals for all evaluation metrics improves the interpretability and reliability of model performance assessment, as highlighted in the recent literature on interpretable radiomics-based prediction and immune signature modeling [16,17]. Our study represents a new use of chest CT imaging data in the prediction of SP recurrence. We employed machine learning algorithms to predict recurrence and simultaneously verify the association between blebs or bullae and SP recurrence. This approach yielded excellent performance from our models, suggesting a potential expansion of the tools available for managing this condition. Through further refinement, these models could achieve more reliable and precise outcomes, which would enhance the role of chest CT imaging data as a valuable predictive tool [12,27]. To the best of our knowledge, this is the first study in which SP recurrence was investigated using machine learning algorithms with chest CT imaging data. While our initial findings are excellent, further research is necessary to validate them. The development of more robust predictive models for SP recurrence—which could lead to improved patient management and outcomes—could be achieved by integrating machine learning algorithms with multi-center data.

In addition to large-scale, multi-center studies, several specific research directions could further strengthen the clinical utility of this approach. A prospective long-term follow-up program extending beyond five years would allow a more complete characterization of recurrence patterns and facilitate evaluation of the predictive value of apical changes or tiny blebs that may develop into more definitive lesions. Such a program could include scheduled chest CT scans at regular intervals (e.g., annually) combined with standardized clinical assessments to track morphological changes over time. Furthermore, a multi-center research plan involving collaboration among tertiary referral hospitals could be implemented to collect and share chest CT datasets from diverse populations. Establishing a centralized imaging repository would increase both sample size and heterogeneity, enabling robust external validation of predictive models. Lastly, incorporating multi-slice or volumetric CT analysis rather than relying solely on a single representative apical image may provide a more comprehensive understanding of lesion distribution and morphology. This could enhance model performance, particularly in cases with lateral or basal blebs that are not well-visualized in a single apical slice.

The present study had several limitations. First, there is a lack of generalizability because of its retrospective nature and restriction to a single hospital. We believe that integrating machine learning algorithms with multi-center data could be extended to develop robust predictive models in the field of SP recurrence, potentially improving patient management and outcomes. Second, these predictive models have limitations when blebs or bullae are located in the lateral or basal regions of the lung; however, a more robust model could be developed by incorporating and training with more cases involving such scenarios or by including multiple sections of chest CT scans rather than relying on a single apical image. Although technically challenging and not feasible in this study, this approach could enhance the model’s generalizability and improve its predictive performance. Third, the chest CT images used were influenced by evolving various medical conditions, potentially affecting the prediction performance for SP recurrence. To mitigate this, strict exclusion criteria were applied, and the observation period was restricted to two years to minimize time-related biases. Fourth, the dataset used in this study was relatively small. To address this limitation, pre-trained transfer modeling and data augmentation techniques were applied, enhancing the applicability of the machine learning model in clinical practice [12].

## 5. Conclusions

The findings of this study demonstrate that machine learning algorithms using chest CT images exhibit excellent performance in predicting SP recurrence in young adolescents without surgery. Furthermore, consistent with clinical knowledge, this study provides direct visual evidence that the presence of blebs or bullae is strongly associated with SP recurrence, while apical regions play a critical role in recurrence even in cases without definitive blebs or bullae. This machine learning approach provides a convenient and valuable tool to support surgical decision-making. However, further large-scale, multi-center studies incorporating multi-level scans are required to validate these findings and develop comprehensive management guidelines for SP. Such multi-center collaborations would be crucial to verify the generalizability of the model and ensure its applicability in diverse clinical settings.

## Figures and Tables

**Table 1:** Baseline clinical characteristics of adolescents with and without recurrence of primary spontaneous pneumothorax, stratified by laterality.

Variables	Right Side			Left Side		
	NRG (*n* = 110)	RG (*n* = 54)	*p*-Value	NRG (*n* = 92)	RG (*n* = 43)	*p*-Value
Age	17.3 ± 1.6	16.9 ± 1.3	0.080	17.5 ± 1.5	17.4 ± 1.5	0.561
Sex (male)	104	52	1.000	85	39	0.743
Smoking	10	5	1.000	6	2	1.000
Blebs or bullae	10	14	0.006	4	14	<0.001

NRG, non-recurrent group; RG, recurrent group.

**Table 2:** Multivariate logistic regression analysis of risk factors for recurrence of spontaneous pneumothorax.

Variables	Odds Ratio	*p*-Value	95% CI
Age	0.823	0.027	0.692–0.978
Blebs or bullae	6.035	<0.001	2.951–12.339

CI, confidence interval.

**Table 3:** Performance of machine learning models in predicting recurrence of primary spontaneous pneumothorax by laterality.

Lung Laterality	Model Type	AUC (95% CI)	Accuracy (95% CI)	F1 Score (95% CI)	Precision (95% CI)	Recall (95% CI)
Right	Neural network	0.970 (0.961–0.979)	0.937 (0.945–0.971)	0.936 (0.918–0.957)	0.937 (0.906–0.962)	0.937 (0.906–0.962)
	Logistic regression	0.958 (0.949–0.967)	0.928 (0.938–0.966)	0.927 (0.908–0.949)	0.928 (0.895–0.954)	0.928 (0.895–0.954)
	Support vector machine	0.950 (0.941–0.959)	0.902 (0.919–0.950)	0.903 (0.877–0.925)	0.904 (0.867–0.934)	0.902 (0.863–0.931)
	Gradient boosting	0.934 (0.925–0.943)	0.868 (0.894–0.930)	0.862 (0.840–0.896)	0.871 (0.828–0.905)	0.868 (0.825–0.902)
	Random forest	0.865 (0.856–0.874)	0.813 (0.858–0.900)	0.798 (0.781–0.848)	0.821 (0.775–0.862)	0.813 (0.765–0.853)
Left	Neural network	0.958 (0.949–0.967)	0.905 (0.919–0.954)	0.905 (0.874–0.928)	0.905 (0.860–0.936)	0.905 (0.860–0.936)
	Logistic regression	0.936 (0.927–0.945)	0.881 (0.903–0.941)	0.881 (0.849–0.909)	0.881 (0.834–0.917)	0.881 (0.834–0.917)
	Support vector machine	0.934 (0.925–0.943)	0.877 (0.900–0.939)	0.877 (0.829–0.914)	0.877 (0.829–0.914)	0.877 (0.829–0.914)
	Gradient boosting	0.907 (0.898–0.916)	0.844 (0.877–0.920)	0.838 (0.807–0.875)	0.843 (0.791–0.884)	0.844 (0.795–0.887)
	Random forest	0.848 (0.839–0.857)	0.786 (0.837–0.886)	0.771 (0.745–0.823)	0.783 (0.729–0.832)	0.786 (0.732–0.835)

AUC, area under the receiver operating characteristic curve; F1 score, harmonic mean of precision and recall; CI, confidence interval. All values are presented as proportions unless otherwise noted.

## Data Availability

Raw data were generated by the Department of Thoracic Surgery at Uijeongbu Saint Mary’s Hospital. The derived data supporting the findings of this study are available from the corresponding author upon reasonable request.
